# Biological Versus Synthetic Grafts in ACL Reconstruction: A Comparative Analysis of Failure Rates, Knee Stability, and Functional Outcomes

**DOI:** 10.15388/Amed.2025.32.2.13

**Published:** 2025-12-30

**Authors:** Karolis Strašunskas, Rokas Jurkonis

**Affiliations:** 1 Lithuanian University of Health Sciences, Faculty of Medicine, Kaunas, Lithuania Karolis95str@gmail.com; 2 Department of Orthopaedic Surgery, Hospital of Lithuanian University of Health Sciences Kaunas Clinics, Kaunas, Lithuania rokas.jurkonis@lsmu.lt

**Keywords:** Anterior cruciate ligament, ACL reconstruction, graft failure, knee stability, functional outcomes, biological graft, synthetic ligament, priekinis kryžminis raištis, PKR rekonstrukcija, transplantacijos nesėkmė, kelio stabilumas, funkciniai rezultatai, biologinis transplantatas, sintetinis raištis

## Abstract

**Background:**

Anterior cruciate ligament (ACL) injuries are among the most common and impactful musculoskeletal injuries, particularly in athletic populations. A variety of biological and synthetic grafts are used in surgical reconstruction, each offering different biomechanical properties and long-term outcomes.

**Materials and Methods:**

This narrative review analyzed 42 original clinical studies published between 1989 and 2024. Articles were retrieved from *PubMed* and *Google Scholar* by using terms such as ‘ACL reconstruction’, ‘BPTB’, ‘hamstring tendon’, ‘quadriceps tendon’, ‘LARS’, ‘Leeds-Keio’, and ‘GORE-TEX’. Key outcomes included graft failure rates, postoperative knee stability (e.g., KT-1000, Lachman test), and functional outcomes (Lysholm, Tegner, IKDC). Numerical data were pooled descriptively; no formal meta-analysis was performed.

**Results:**

Biological grafts, particularly BPTB and quadriceps tendon autografts, demonstrated the lowest failure rates (as low as 1.2%) and superior mechanical stability (>95% achieving Grade 0–1 laxity) compared to synthetic options. Hamstring tendon autografts were slightly less durable but still reliable. Synthetic grafts, and especially GORE-TEX and Leeds-Keio, were associated with higher failure rates (up to 33%) and complications related to poor biological integration.

**Conclusions:**

BPTB and QT autografts remain the most reliable options for ACL reconstruction, offering excellent long-term outcomes. While synthetic grafts may be appropriate in select patients, they carry a higher risk of failure and complications. Graft selection should be individualized based on the patient activity level, anatomical considerations, and tolerance for donor-site morbidity.

## Abstract (it)

**Įvadas:**

Priekinio kryžminio raiščio (PKR) traumos yra vienos iš dažniausių ir labiausiai turinčių įtakos raumenų ir kaulų sistemos traumų, ypač sportuojančių žmonių. Chirurginei rekonstrukcijai naudojami įvairūs biologiniai ir sintetiniai transplantatai, pasižymintys skirtingomis biomechaninėmis savybėmis ir ilgalaikiais rezultatais.

**Medžiaga ir metodai:**

Šioje apžvalgoje buvo analizuoti 42 originalūs klinikiniai tyrimai, paskelbti nuo 1989 iki 2024 metų. Straipsniai pasirinkti iš *PubMed* ir *Google Scholar*, vartojant tokius raktažodžius, kaip antai „PKR rekonstrukcija“, „BPTB (kaulas–kelio girnelė–kaulas)“, „pakinkčio raištis“, „keturgalvio raumens raištis“, „LARS“, „Leeds-Keio“ ir „GORE-TEX“. Vertinta informacija apie transplantacijos nesėkmių dažnį, pooperacinį kelio stabilumą (pvz., KT-1000, Lachmano testas) ir transplantatų naudojimo funkcinius rezultatus (Lysholm, Tegner, IKDC). Skaitmeniniai duomenys buvo apibendrinti ir aprašyti; formali metaanalizė nebuvo atlikta.

**Rezultatai:**

Biologiniai transplantatai, ypač BPTB ir keturgalvio raumens sausgyslių autotransplantatai, pasižymėjo mažiausiu jų naudojimo nesėkmių dažniu (tik 1,2 %) ir pranašesniu mechaniniu stabilumu (daugiau nei 95 % pasiekė 0–1 laipsnio laisvumą), palyginti su sintetiniais variantais. Kojos raumens sausgyslių autotransplantatai buvo įvertinti kaip šiek tiek mažiau patvarūs, bet vis tiek patikimi. Sintetinių transplantatų, ypač GORE-TEX ir Leeds-Keio, naudojimas buvo susijęs su didesniu nesėkmių dažniu (iki 33 %) ir komplikacijomis dėl prastos biologinės integracijos.

**Išvados:**

BPTB ir QT autotransplantatai išlieka patikimiausiu pasirinkimu ACL rekonstrukcijai, užtikrina puikius ilgalaikius rezultatus. Nors sintetiniai transplantatai gali būti tinkami tam tikriems pacientams, jie kelia didesnę nesėkmės ir komplikacijų riziką. Transplantatas turėtų būti parenkamas individualiai, atsižvelgiant į paciento aktyvumo lygį, anatominius ypatumus ir toleranciją donorinės vietos komplikacijoms.

## Introduction

### Anatomy and Function

The *Anterior Cruciate Ligament* (ACL) is a dense band of connective tissue that provides critical stability to the knee joint by connecting the femur to the tibia. It is intra-articular but extra-synovial, originating from the medial surface of the lateral femoral condyle and inserting into the medial tibial eminence, running in a distal–anterior–medial direction [1]. Structurally, the ACL consists of two distinct bundles: the *Anteromedial* (AM) bundle, which becomes tight during knee flexion, contributing significantly to anterior stability and rotational control, and the *Posterolateral* (PL) bundle, which tightens during knee extension, providing stability predominantly in extension [1], [2].

The ACL width ranges from 7 to 12 mm, and its length varies between 22 and 41 mm. Its femoral and tibial insertions are more than three times wider than the midsubstance, highlighting the necessity for precise tunnel placement in ACL reconstruction to ensure graft integration and prevent failure or impingement [1], [2]. The ligament has limited vasculartization, primarily from the middle genicular artery, contributing to poor healing potential at tis avascular insertion sites [1]. Moreover, the ACL contains proprioceptive mechanoreceptors crucial for joint position awareness. Injuries to the ACL disrupt this proprioceptive feedback, resulting in knee instability. Thus, preserving ACL remnants during reconstruction may maintain the proprioceptive function, influencing muscle activation patterns, particularly in the hamstring [1].

Functionally, the ACL prevents excessive anterior tibial translation, hyperextension, and abnormal rotational movements. Its tension varies with the knee flexion angle: during extension, the PL bundle is primarily under tension, while the AM bundle tightens during flexion [1]. Single-bundle ACL reconstruction, which replaces the torn ligament with a single graft positioned to restore both bundles’ combined function, remains the most widely performed surgical approach and is the focus of this review [2], [3], [5].

### ACL Injury Mechanisms

ACL injuries predominantly occur through non-contact mechanisms, often during athletic movements involving sudden landing, cutting, or pivoting. Common kinematic risk factors include knee valgus alignment, internal tibial rotation, reduced hip and knee flexion upon landing, and a high quadriceps-to-hamstring activation ratio. Neuromuscular deficits, such as inadequate hip control and delayed hamstring activation, also contribute to injury risk. Approximately 70–80% of ACL injuries occur without direct contact, thereby emphasizing the importance of neuromuscular training and jump-landing programs, which have shown a 50% reduction in ACL injuries, particularly in young athletes [3], [4].

### Diagnosis and Risk Factors

Clinically, ACL injuries typically present with persistent knee effusion, instability, and a characteristic ‘pop’ at the injury event. The Lachman test remains the most accurate clinical assessment tool, whereas the pivot shift test has high specificity but lower sensitivity, especially in acute cases. The anterior drawer test is more sensitive for chronic injuries [3]. Imaging studies include initial radiographs to exclude fractures, followed by MRI, the gold standard, with an accuracy between 82% and 100% for ACL tear diagnosis. The Lever sign test also demonstrates potential with reported sensitivity and specificity nearing 100%, though further validation is needed [3].

### Epidemiology and Risk Factors

ACL injuries constitute a significant health issue, with over 120,000 cases occurring annually in the United States alone. Incidence continues to rise, particularly among female athletes. Knee injuries represent 60% of all sports-related injuries, with approximately half involving the ACL, contributing to an annual expenditure of roughly $1 billion in surgical management [3]. Female athletes have a 2.1- to 3.4-fold higher risk of sustaining ACL injuries compared to males, particularly in high-risk sports like soccer (0.8%) and basketball (0.4%) [3]. Around 41% of ACL injuries occur via a non-contact mechanism, thus underscoring the importance of preventive strategies. Overall, about 250,000 ACL injuries occur annually, with roughly 100,000 surgical reconstructions performed each year [3].

Notably, patients undergoing ACL reconstruction carry a significant risk of graft rupture or contralateral ACL injury within two years. Smaller graft diameters have been linked to an increased graft failure risk [3]. The long-term financial burden is substantial, averaging $38,121 per surgical patient compared to $88,538 for those managed non-operatively over their lifetime [4]. Furthermore, approximately 50% of ACL-injured patients develop *Osteoarthritis* (OA) within 10–20 years post-injury, irrespective of the treatment choice [4].

Known anatomical risk factors for ACL injuries include a narrow femoral notch width, smaller ACL cross-sectional area, increased posterior tibial slope, and generalized ligamentous laxity, especially in female athletes. Additional factors include excessive foot pronation, genu recurvatum, and a high *Body Mass Index* (BMI). Females also demonstrate biomechanical risk factors such as reduced hip and knee flexion angles during landing, an increased knee valgus, and heightened quadriceps-to-hamstring activation ratios [4]. Hormonal influences, particularly estrogen and progesterone, may further affect ACL susceptibility, although definitive associations with the menstrual cycle remain unclear. External factors like shoe design with higher torsional resistance, artificial playing surfaces, and synthetic floors increase ACL injury risk, whereas colder climates may reduce it [4]. Genetic predisposition, involving genes like COL1A1, COL5A1, MMP10, and MMP3, also influences ACL rupture susceptibility. Additionally, individuals with previous ACL reconstruction face a 15% higher risk of graft rupture compared to primary tears, with graft rupture rates ranging from 6% to 32% depending on neuromuscular function, trunk control, and postoperative activity levels [4].

### Surgical Considerations in ACL Reconstruction

ACL reconstruction surgery aims to restore native knee biomechanics, minimize graft failure, and promote long-term stability. Critical surgical objectives include precise anatomical graft placement, restoration of both AM and PL bundle functions, and robust graft fixation to support early integration and rehabilitation [1], [2], [5]. Correct tunnel positioning, particularly at the femoral insertion, is crucial; even minor deviations can significantly affect graft tension, potentially leading to impingement, instability, or graft failure [5], [6].

Graft isometry – maintaining consistent tension throughout the knee’s range of motion – is essential, particularly for synthetic grafts, which are more vulnerable to fatigue from repeated length changes [6]. Regardless of graft type, optimal fixation in dense cortical bone and replication of the ACL’s native orientation can improve biomechanical performance, limit tunnel widening, and reduce graft laxity over time [5].

Early complications following ACL reconstruction, such as infection, hemarthrosis, effusion, or stiffness, are generally related to surgical technique or postoperative rehabilitation protocols [41]. Late complications, including graft rupture, persistent laxity, tunnel widening, synovitis, and progressive osteoarthritis, are more closely linked to graft material properties and long-term biological integration [34], [41].

### Purpose of the Review

The purpose of this narrative review is to compare the clinical performance of biological (*Bone-Patellar Tendon-Bone* [BPTB], *Hamstring Tendon* [HST], *Quadriceps Tendon* [QT]) and synthetic (LARS, Leeds-Keio, GORE-TEX) grafts used in single-bundle anterior cruciate ligament (ACL) reconstruction. The review focuses on three core outcome domains: (1) graft failure rates, (2) postoperative knee stability, and (3) long-term functional outcomes. Additionally, the review distinguishes between early postoperative complications, primarily influenced by the surgical technique, and late complications, which are more closely linked to the graft material and biological integration. The aim is to guide clinicians toward evidence-based graft selection tailored to the patient’s activity level, anatomical factors, and tolerance for donor-site morbidity.

## Materials and Methods

### Search Design

This review was conducted as a structured narrative review with the primary objective of comparing biological and synthetic grafts used in anterior cruciate ligament (ACL) reconstruction. The focus was on evaluating the graft failure rates, postoperative knee stability, and long-term functional outcomes across major graft types, including bone-patellar tendon-bone (BPTB), hamstring tendon (HST), quadriceps tendon (QT), and synthetic options such as LARS, Leeds-Keio, and GORE-TEX.

### Databases and Search Terms

- PubMed
- Google Scholar

Searches were performed using the following key terms individually and in combination with Boolean operators (AND, OR):

- ‘ACL reconstruction’
- ‘Bone-patellar tendon-bone’ OR ‘BPTB graft’
- ‘Hamstring tendon graft’
- ‘Quadriceps tendon ACL’
- ‘LARS ligament’
- ‘Leeds-Keio ligament’
- ‘GORE-TEX ACL graft’

The reference list of included articles was also reviewed by using a snowballing technique to capture additional relevant studies. Only articles published in English were considered.

### Inclusion Criteria

Studies were included if they met the following criteria:

- Original clinical research (retrospective, prospective cohort, RCTs) involving human subjects.
- Evaluated ACL reconstruction using biological (BPTB, HST, QT) or synthetic (LARS, Leeds-Keio, GORE-TEX) grafts.
- Reported at least one of the following outcomes:
  - Graft failure rate (rupture, revision)
  - Postoperative knee stability (KT-1000, Lachman, pivot shift)
  - Functional outcomes (Lysholm, IKDC, Tegner)
- Provided quantitative data or comparative findings.
- Published in peer-reviewed journals in English.

### Exclusion Criteria

- Biomechanical, animal, cadaveric, or in vitro studies without clinical outcomes.
- Case reports, conference abstracts, editorials, or reviews.
- Studies lacking outcome data on failure, stability, or function.
- Articles not available in the full-text format.

### Data Extraction and Synthesis

Relevant data were extracted from studies according to predefined categories:

- Study characteristics: authorship, year, graft type, sample size, and follow-up duration.
- Primary outcomes: graft failure rates, revision frequency.
- Secondary outcomes: KT-1000/laxity measurements, Lachman and pivot shift grades, Lysholm, IKDC, and Tegner scores.
- Postoperative complications or integration issues (e.g., tunnel widening, synovitis).

As this was a narrative review, no formal quality scoring tool (e.g., Cochrane RoB) was applied. Nonetheless, studies were evaluated for methodological soundness, clinical relevance, and consistency of the reported findings. The data were synthesized narratively, and numerical values (including those shown in tables and figures) represent pooled or averaged estimates based on convergence across studies.

To facilitate clinically relevant comparisons, outcomes were synthesized and reported separately for each graft type (BPTB, HST, QT, LARS, Leeds-Keio, GORE-TEX), rather than organized solely by the outcome category. This approach enabled clear evaluation of graft-specific performance across failure rates, mechanical stability, and functional outcomes.

## Results

### Bone-Patellar Tendon Bone (BPTB)

#### Graft Failure Rates

**Figure 1:**
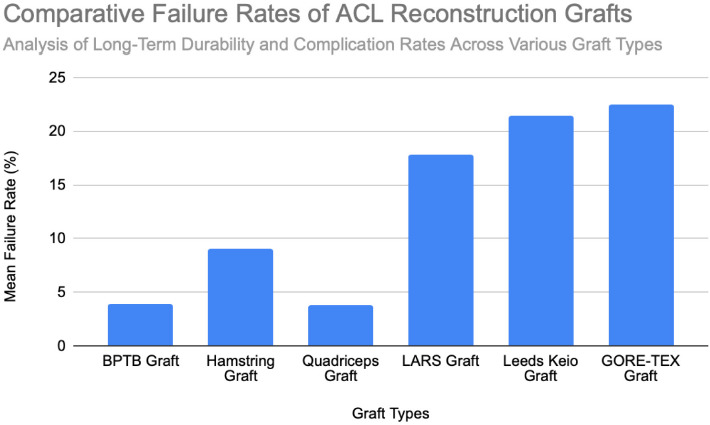
Mean failure rates and variability across biological (BPTB, HST, QT) and synthetic (LARS, Leeds-Keio, GORE-TEX) grafts in ACL reconstruction. Data derived from clinical studies involving sample sizes ranging from n = 26 to n = 7,560 patients per graft type. Chart created by the author.

The BPTB autograft has consistently demonstrated excellent long-term durability in ACL reconstruction, with pooled failure rates around 3.9% [Figure 1]. A 15-year survival rate of 94.8% has been reported, though an early graft rupture may still occur, with failure rates of up to 8.2% during the first postoperative year [7]. Over long-term follow-up, revision rates remain low; only 2% of patients required reoperation over 30 years, and the reported rupture rates across clinical series average approximately 3.5%, with values ranging from 1.2% to 5.6% [9], [10], [11], [12]. The primary causes of graft failure in BPTB reconstructions are typically traumatic re-injury, particularly during an early return to sport, and technical errors such as malpositioned femoral tunnels which increase the graft stress. One registry-based study involving 7,560 cases showed a rupture rate of 2.8%, reinforcing the procedure’s long-term reliability [10]. A separate report of multiple reoperations following trauma involved synthetic revision grafts, underscoring the continued use of BPTB as a salvage option [13]. Overall, failures in BPTB grafts are most commonly related to a trauma or the surgical technique, rather than inherent graft fragility, thereby affirming their status as a gold-standard option for mechanically durable ACL reconstruction.

#### Knee Stability

**Figure 2:**
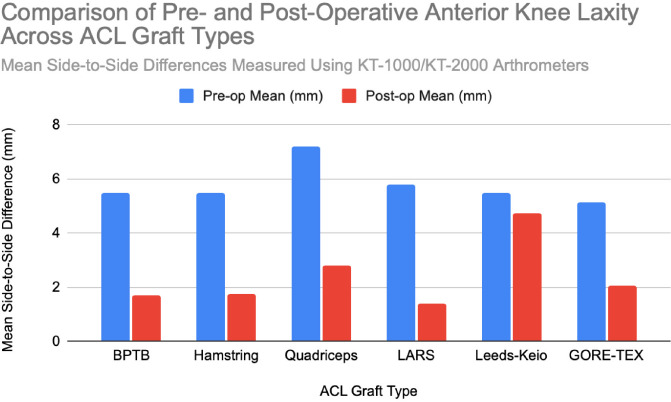
Instrumented laxity measurements (KT-1000/2000) indicating side-to-side anterior translation following ACL reconstruction. Pooled data from studies with n = 20 to n = 7,560 patients across six graft types. Chart created by the author.

At six months postoperatively, 86% of patients demonstrated acceptable knee stability (<3mm side-to-side difference); however, this declined to 68% at 3–6 years. At that time, 8% exhibited laxity exceeding 5 mm, representing a slight but statistically significant increase in laxity over time [14]. At a final follow-up (mean 61.8 ± 25.9 months), the mean anterior translation difference was 1.4 ± 2.1 mm, indicating preserved long-term mechanical stability [11]. Lachman testing at five years revealed minimal-to-mild laxity in most patients, with no significant differences between autograft and synthetic groups [13]. Positive Lachman tests were observed in 25% of surviving grafts, and pivot shift tests in 19% [10]. Even at 30 years postoperatively, only one patient demonstrated a grade 2–3 pivot shift, and three patients showed grade 2–3 Lachman results [8]. Instrumented testing revealed ≥3 mm laxity in 22% of surviving BPTB grafts [10]. Notably, an increased postoperative laxity was predominantly attributed to femoral tunnel malposition rather than graft elongation. Among seven patients with KT-1000 side-to-side differences exceeding 3 mm, five had incorrectly placed femoral tunnels, thereby underscoring the importance of surgical precision for long-term outcomes [14]. At 15-year follow-up, 90% of patients had returned to their pre-injury activity levels. Subjective satisfaction was high, with 98.1% reporting positive outcomes. Objective assessments further supported functional success: 93.5% demonstrated normal pivot shift results, and 95.4% achieved normal Lachman grades based on KT-1000 testing [7]. Overall, more than 95% of patients achieved Grade 0 or 1 laxity. Additionally, 72.2% maintained <3 mm anterior translation on KT-1000 testing, thus reinforcing the BPTB autograft’s reputation for long-term structural reliability [12], [8]. BPTB graft stability is summarized in [Figure 2].

#### Functional Outcomes

**Figure 3:**
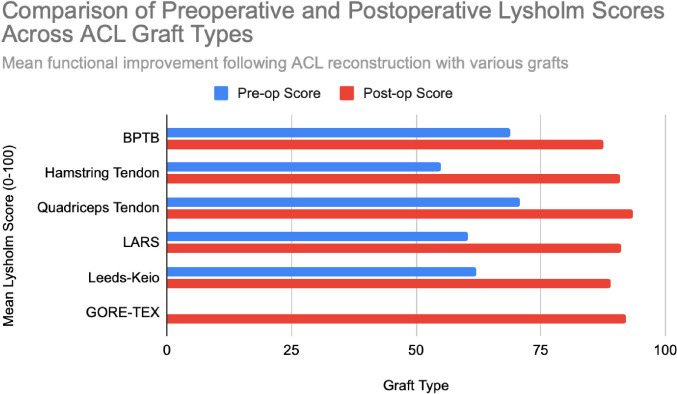
Mean Lysholm functional scores reported in clinical cohorts following ACL reconstruction. Data based on studies with n = 25 to n = 198 patients per graft type. Chart created by the authors.

**Figure 4:**
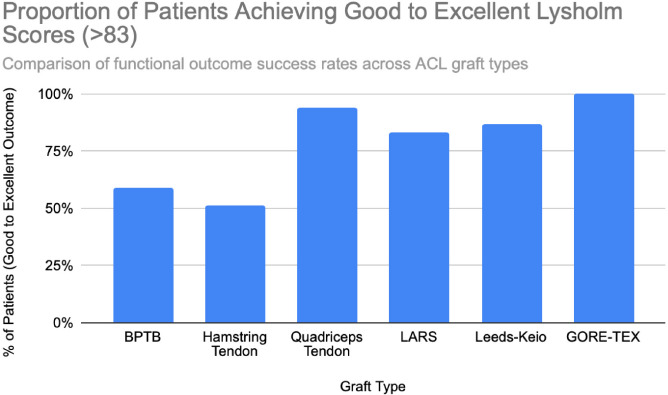
Distribution of Lysholm scores by outcome category (good ≥83, excellent ≥95) across graft types. Data based on study populations ranging from n = 22 to n = 198 patients. Chart created by the authors.

**Figure 5:**
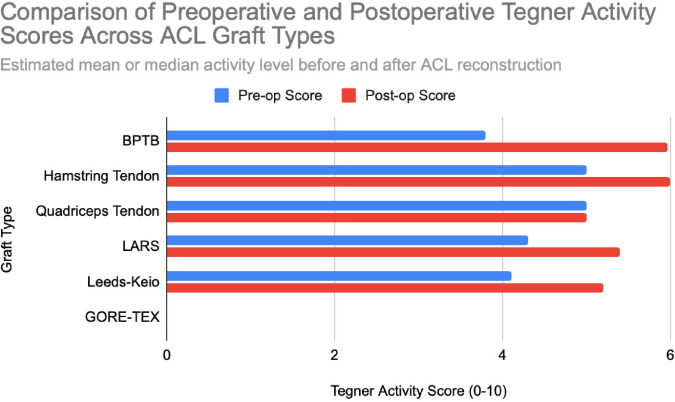
Representative pre- and postoperative Tegner activity levels for each graft type. Values extracted from studies with n = 25 to n = 188 patients. Chart created by the authors.

**Figure 6:**
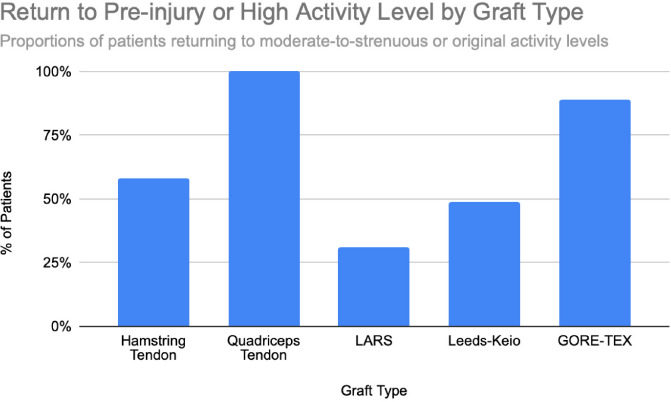
Percentages or patients returning to sport or pre-injury activity level following ACL reconstruction. Estimates derived from studies with sample sizes ranging from n = 26 to n = 235 per graft type. Chart created by the authors.

**Figure 7:**
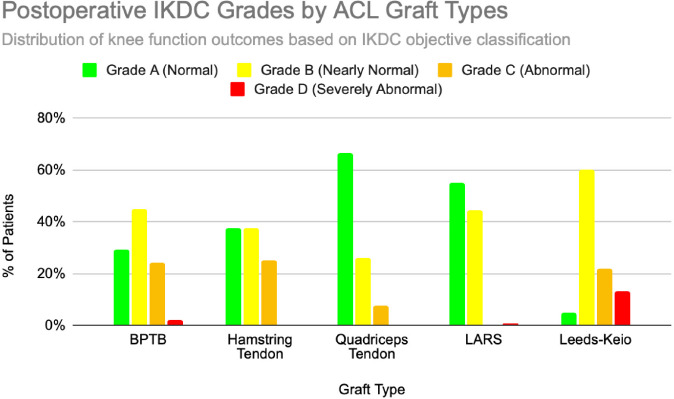
Postoperative distribution of IKDC grade (A: normal, B: nearly normal, C: abnormal, D: severely abnormal) by graft type. Data extracted from studies involving n = 18 to n = 198 patients. Charts created by the authors.

Functional outcomes following ACL reconstruction showed consistent improvement across standardized measures such as Lysholm, Tegner, and IKDC scores. In the synthetic graft group, Lysholm scores increased significantly, peaking at 90 at one year and remaining stable at 87 at five years, with 52% of patients achieving ‘excellent’ outcomes (scores ≥95) [13]. In the BPTB group, results were particularly robust: Lysholm scores improved from a preoperative mean of 69.2 to 91.5, with 59% of patients achieving good to excellent outcomes (Lysholm ≥83) [8], [9], [Figure 3], [Figure 4]. IKDC assessments at 3–6 years showed 29% of patients rated as ‘normal’, 45% as ‘nearly normal’, 24% as ‘abnormal’, and 2% as ‘severely abnormal’. Additionally, 80% of patients reported stable knees, while only 4% experienced significant subjective instability [14], [Figure 7]. Tegner activity scores also improved substantially postoperatively, rising from a preoperative mean of 3.8 to 4.1 at six months, 5.2 at one year, and stabilizing at approximately 6.2 at two and five years [13]. In a dedicated BPTB cohort, the mean postoperative Tegner level reached 8.1 with a greater proportion of patients returning to their pre-injury Tegner level of 9 [9], [Figure 5]. Regarding return-to-sport outcomes, athletes receiving BPTB autografts demonstrated a 74.6% return-to-sport rate, typically occurring at around 9.7 months post-surgery [9]. At a final follow-up (mean 61.8 ± 25.9 months), 79% of patients had resumed sporting activities, with 40% returning to their exact pre-injury activity level [11], [Figure 6]. These findings highlight the functional strength and return-to-activity potential of BPTB grafts, underscoring the consistency of long-term outcomes among biological graft types.

### Hamstring Tendon

#### Graft Failure Rates

*Hamstring Tendon* (HST) autografts generally yield favorable outcomes in ACL reconstruction, although failure rates vary based on the graft configuration, patient characteristics, and the surgical technique. In revision ACL reconstructions, failure rates average approximately 10%, with most ruptures occurring at around 46 months postoperatively [15]. Importantly, 65% of these failures were attributed to traumatic reinjury, thereby indicating that most failures reflect patient activity and the surgical context rather than intrinsic graft weakness [15]. In primary ACL reconstructions, the reported rupture rates range from 2.84% at 5.6 years to 9% at 14.6 years postoperatively, with an additional 5% experiencing contralateral ACL ruptures [10], [17]. Higher rupture rates (up to 22.1%) have been observed in athletic populations and are commonly associated with smaller graft diameters (<8 mm) or suboptimal tendon-to-bone healing [16]. Reinjury in the early postoperative period also remains a concern, with failure occurring as early as 10.7 months post-surgery in approximately 7% of patients, typically following high-impact activity [17]. Additionally, around 4% of patients developed increased laxity or a positive pivot shift test without a clear traumatic event, thereby suggesting potential graft elongation or biomechanical insufficiency [17]. Graft configuration influences its durability: semitendinosus-gracilis grafts with preserved insertions (STGPI) exhibit markedly lower failure rates (1.2%) compared to free tendon constructs (STGF), which demonstrate rates of approximately 7.1% [11]. Taken together, these findings support the reliability of HST autografts, though a small graft diameter, high activity levels, and technical factors may elevate the failure risk. The overall mean failure rate across studies is approximately 9% [Figure 1].

#### Knee Stability

Instrumented KT-1000 measurements demonstrated a mean anterior tibial translation of 7.2 mm in reconstructed knees versus 6.2 mm in non-reconstructed knees, which is a statistically significant difference, though likely not clinically meaningful [16]. Stability assessments in revision cases were favorable: Lachman testing indicated <3 mm laxity in 53% of patients, mild laxity (3–5 mm) in 45%, and severe laxity (>5 mm) in only 2% [15]. Pivot shift tests were negative in 69% of these cases, with mild instability in the remaining 31%. Mean KT-1000 laxity difference was 2.5 mm, with 50% of patients maintaining <3 mm laxity [15]. Long-term evaluations showed sustained outcomes: 76% of patients demonstrated ≤3 mm translation, while 11% had mild laxity (3.1–5.0 mm), and 6% exhibited moderate laxity (>5 mm) [17]. Lachman and pivot shift tests were negative in 89% of patients, thus indicating an overall good knee stability despite mild residual laxity in a subset [17]. A meta-analysis reported instrumented laxity in 18% of surviving hamstring grafts, with positive Lachman and pivot shift tests in 25% and 17%, respectively [10]. Collectively, these findings support hamstring autografts as effective and reliable options for maintaining long-term knee stability after ACL reconstruction. A comparative visual summary of hamstring graft stability outcomes is presented in [Figure 2].

#### Functional Outcomes

Hamstring autografts consistently demonstrate significant postoperative improvements in both functional scores and subjective outcomes, though long-term performance often remains slightly below pre-injury levels. IKDC ratings show that 73–75% of patients report normal or nearly normal knee function, while approximately 25–27% fall into abnormal or severely abnormal categories [15], [16], [Figure 7]. Lysholm scores improved significantly, rising from a preoperative mean of 55 to postoperative means ranging from 76.7 to 91, with 47–56% of patients achieving good to excellent outcomes (scores >83) [15], [16], [17], [Figure 3], [Figure 4]. Tegner activity levels exhibited statistically significant reductions post-surgery, decreasing from a median score of 7 (competitive sports) to approximately 5 (recreational sports). Despite this decline, most patients resumed moderate to strenuous activities [15], [16], [17], [Figure 5]. In revision ACL reconstructions, 58% of patients returned to moderate-to-strenuous activity (Levels 1–2), while 42% remained limited to lighter or sedentary activities (Levels 3–4) [15]. Patient-reported outcomes supported the overall functional success: 71% reported being pain-free during strenuous activity, 82% had no swelling, and 91% reported no instability. However, 44% experienced moderate kneeling pain (mean intensity 5/10), reflecting a known drawback of hamstring graft harvesting [15]. Quality-of-life assessments (ACL-QOL) showed an average satisfaction rate of approximately 67%. Still, 52% of patients did not return to their previous sports and only 5% resumed contact sports [16]. Among athletes specifically, 53.5% returned to their pre-injury performance level at a mean of 10.5 months postoperatively [9], [Figure 6]. Collectively, these findings support the functional effectiveness of hamstring autografts, while acknowledging the variability in return-to-sport outcomes and the influence of individual patient factors on long-term recovery.

### Quadriceps Tendon

#### Graft Failure Rates

*Quadriceps Tendon* (QT) grafts demonstrate favorable outcomes in ACL reconstruction, with a mean failure rate of approximately 3.8%, ranging from 0.0% to 6.0% across clinical series [18], [19], [20]. These findings highlight the graft’s mechanical reliability and low complication profile. In one series of 198 reconstructions, 6% of patients required revision ACL surgery due to graft failure, indicating a traumatic rupture sustained during a soccer match at 45 months, and three atraumatic failures occurring at 24, 26, and 30 months, possibly linked to technical errors or postoperative noncompliance [18]. Another study involving 57 patients reported zero graft ruptures or quadriceps tendon failures at a 2-year follow-up [20]. Notably, no patellar fractures occurred in patients receiving bone block–harvested QT grafts, thus confirming the structural safety of this technique. One isolated case of a patellar fracture was reported at five months postoperatively, though it was managed conservatively without long-term complications [19]. Across studies, no patients developed a major extensor mechanism dysfunction or significant donor-site morbidity, thereby reinforcing the status of QT autografts as a durable and safe option for ACL reconstruction. Comparative failure rates for all graft types are shown in [Figure 1].

#### Knee Stability

Quadriceps tendon autografts consistently provide excellent postoperative knee stability. Clinical assessments show that over 95% of patients achieved Grade 0 or 1 stability based on Lachman, anterior drawer, and pivot shift tests [12], [18]. Arthrometric testing using the KT-2000 demonstrated a reduction in the mean knee laxity from 7.2 mm preoperatively to 2.1 mm postoperatively, with 75% of patients exhibiting less than 2 mm of side-to-side difference [18]. Long-term follow-up confirmed that 94% of patients maintained anterior translation under 5 mm, thus representing a substantial improvement from severe preoperative laxity [21]. Stress radiographs further demonstrated anterior tibial translation improvements from 5.4 mm preoperatively to 1.0 mm postoperatively. Additionally, complete resolution of positive pivot shift tests was observed by 12 months, thus supporting the graft’s effectiveness in restoring both anterior-posterior and rotational stability [22]. A visual summary of these outcomes is provided in [Figure 2].

#### Functional Outcomes

Quadriceps tendon autografts have demonstrated excellent functional recovery in ACL reconstruction, with significant improvements in Lysholm, Tegner, and IKDC scores. Lysholm scores improved from preoperative ranges of 61.4 to 71 to postoperative scores of 90 to 93.4, with up to 94% of patients achieving good or excellent outcomes [12], [18], [21]. In a more recent study, Lysholm scores returned to pre-injury levels, increasing from 89.9 to 93.4 at final follow-up, with all patients reporting satisfactory outcomes [20]. IKDC classifications showed substantial postoperative improvement: preoperatively, 63–68% of patients were rated as ‘abnormal’ whereas, by final follow-up, 66.7–79% had achieved ‘normal’ scores, and fewer than 10% remained in the abnormal range [18], [19], [21], [Figure 7]. Tegner activity levels largely remained stable, with most patients maintaining their pre-injury activity level, including participation in high-impact sports [18], [20], [Figure 5]. Comparative analyses suggest slightly better knee stability and lower failure rates in quadriceps tendon autografts without a bone plug (QT) compared to those with a bone plug (BQT), although these differences were not statistically significant [19]. Additionally, no significant gender-based differences in outcomes were observed, with both male and female patients reporting minimal side-to-side laxity, range of motion loss, and pain [19], [20]. Lysholm outcomes, return-to-sport rates, and functional comparisons are illustrated in [Figure 3], [Figure 4], and [Figure 6]. Collectively, these findings affirm the status of quadriceps tendon autografts as a reliable option for restoring the knee function and supporting return to sports across diverse patient populations.

### LARS

#### Graft Failure Rates

LARS grafts demonstrate highly variable failure rates influenced by the patient activity level, surgical technique, and follow-up duration. Early studies reported relatively low failure rates of approximately 4.4%, with failures most commonly linked to sports-related trauma, tunnel malposition, or mild knee synovitis [22], [28]. Minor complications, such as femoral or tibial hardware loosening, occurred in 1.9% of the cases, and were successfully managed with revision surgery [22]. However, some cohorts have reported higher failure rates – up to 27.8% – due to a combination of sports trauma, atraumatic instability, and deep surgical site infections, thus highlighting the sensitivity of LARS performance to surgical precision and patient selection [23]. In a longer-term follow-up, mechanical failure rates have reached 33.3%, with graft ruptures often occurring at approximately 3.9 years postoperatively during either high-impact activity or non-traumatic movements. Failures were frequently localized at the femoral tunnel aperture, suggesting mechanical fatigue and abrasion as key contributors [24]. In contrast, lower-demand patients, and particularly older individuals, have shown low complication rates and no graft ruptures, underscoring the importance of the appropriate clinical indication [26]. Additional analyses reported failure rates between 4.4% and 6.9%, particularly among patients with higher preinjury activity levels (Tegner >6), though statistical significance was not always reached [28], [29]. Taken together, the mean reported LARS graft failure rate is approximately 17.8%, with a wide range from 0% to 33.3% [Figure 1]. These findings emphasize the mechanical and biological limitations of synthetic grafts, particularly in active populations.

#### Knee Stability

Postoperative knee stability improved significantly following ACL reconstruction with LARS grafts. KT-1000 arthrometer assessments demonstrated a reduction in anterior tibial translation from 5.8 ± 1.1 mm preoperatively to 1.5 ± 1.6 mm postoperatively, with only 3% of patients experiencing residual laxity [22]. Clinical evaluations, including Lachman tests, reflected a similar improvement – as preoperative laxity rates of 31.4% (grade 1+) and 68.6% (grade 2+) decreased to 86.5% of patients showing no laxity, 10.8% with grade 1+, and only 2.7% with grade 2+ postoperatively [22]. Pivot shift tests further demonstrated that 88.5% of patients achieved full rotational stability (grade 0) following surgery [22]. Subgroup analysis using the KT-2000 arthrometer reported mean side-to-side differences of 1.5 mm for intact grafts, 1.3 mm for revised grafts, and 5.3 mm for re-ruptured grafts, thereby highlighting the close association between graft integrity and objective stability outcomes [23]. These findings were corroborated by other studies, which documented reductions in anterior laxity from 5.1 ± 1.3 mm preoperatively to 1.4 ± 1.5 mm postoperatively, with only 2.2% of patients exhibiting >5 mm residual laxity [28]. When intact, LARS grafts maintained near-normal knee stability, with average side-to-side differences of approximately 1.0 mm [24]. Collectively, these data support the effectiveness of LARS grafts in restoring knee stability, particularly in cases where the graft remains intact. For comparative visual summaries, refer to [Figure 2].

#### Functional Outcomes

LARS synthetic grafts have consistently demonstrated substantial functional and subjective improvements following ACL reconstruction. Lysholm scores improved markedly from preoperative means of 54.6–65.1 to postoperative means ranging from 85.4 to 94.5, with up to 50% of patients achieving ‘excellent’ outcomes (scores >90) and 33.3% classified as ‘good’ (scores 84–90) [22], [23], [25], [Figure 3], [Figure 4]. Similarly, IKDC classifications showed dramatic improvements: while 80–100% of patients were rated ‘abnormal’ or ‘severely abnormal’ (Grades C and D) preoperatively, 90-99% achieved “normal” or ‘nearly normal’ status (Grades A or B) postoperatively [22], [27], [29], [Figure 7]. Subjective IKDC scores ranged from 84.5–87.9, with 62.5% of patients rating their outcomes as ‘excellent’ and 37.5% as ‘good’, with no patients reporting ‘fair’ or ‘poor’ outcomes [24], [26], [29]. Tegner activity levels increased from preoperative means of 3.1–5.5 to postoperative levels of 4.7–6.1. Despite this improvement, a mild decline relative to pre-injury activity was noted, particularly among competitive athletes, with only 31.25% returning to their previous Tegner level [23], [26], [28], [Figure 5], [Figure 6]. Subgroup analyses revealed higher Lysholm and IKDC scores among patients with intact LARS grafts compared to those undergoing revision or experiencing graft failure, although these differences were not statistically significant [23]. Overall, 86.9% of patients returned to sports, and patient-reported outcome measures (PROMs), including the SF-36 Physical Component Score (median 94.1), indicated high satisfaction with surgical outcomes [24], [28]. Taken together, LARS grafts offer strong early-to-midterm functional recovery and high patient satisfaction. However, potential risks of reduced sports performance and graft failure underscore the importance of careful patient selection, particularly in high-demand athletic populations.

### Leeds-Keio

#### Failure Rates

Leeds-Keio (LK) synthetic grafts exhibit substantial variability in failure rates, influenced by the graft configuration, surgical technique, and postoperative loading. In the long-term follow-up (mean: 11.9 years), graft rupture occurred in 12% of patients, and 17% required revision surgery due to a graft failure [30]. Most failures were attributed to trauma, graft elongation, or progressive mechanical degradation. The addition of extra-articular reinforcement was associated with reduced clinical instability, and 94% of patients maintained the full knee range of motion [30]. In a separate cohort, 12.5% of patients experienced a graft rupture within two years, largely from sports trauma or graft stretching, while 25% reported persistent knee instability, resulting in a cumulative clinical instability rate of 38% and multiple reoperations [31]. Another long-term evaluation reported a 10% failure rate due to sprain-related injuries and mechanical fatigue, typically requiring revision with BPTB autografts [32]. A study comparing single- versus double-loop configurations reported a high failure rate of 47% at five years in the single-loop group, thus highlighting the importance of the graft design [33]. Conversely, one trial reported a lower revision rate (1 of 24) and moderate graft fraying in 3 cases, suggesting more favorable outcomes when surgical precision and graft tensioning were optimized [13]. In one of the longest available follow-ups (mean: 13.3 years), 28% of patients confirmed a graft rupture, 33% required at least one additional knee surgery, and 11% reported chronic knee pain, which was severe enough to affect their work capacity [34]. Collectively, these findings suggest a mean failure rate of approximately 21.4%, with reported values ranging from 4.2% to 47% [Figure 1]. The wide variation in outcomes reflects persistent concerns regarding the mechanical durability and biological compatibility of Leeds-Keio grafts.

#### Knee Stability

Leeds-Keio grafts demonstrated declining knee stability from early to long-term follow-up. Lachman tests results showed a decrease in grade 0 or 1+ stability from 66% at one year to 38% at five years postoperatively. Concurrently, the proportion of patients with severe laxity (Grades 2+ and 3+) increased to 53%, rising to 62% when including graft ruptures [33]. Pivot-shift test performance also declined, with the proportion of negative results decreasing from 47% at one year to 22% at five years [33]. Instrumented KT-1000 testing corroborated these findings: the proportion of patients with ≤5 mm anterior laxity declined from 68% at one year to 50% at five years, while moderate (6–10 mm, 35%) and severe laxity (>10 mm, 4%) became more prevalent over time [33]. Additional long-term studies confirmed persistent knee laxity, with 22% of patients exhibiting clinically significant anterior translation (>5 mm) [34]. Clinical evaluations revealed ongoing anterior-posterior and rotational instability in a subset of patients during an extended follow-up, particularly among those who received synthetic grafts [34], [35]. Secondary procedures and arthroscopic evidence of graft wear further raised concerns regarding mechanical durability and long-term structural performance [13]. Collectively, these findings highlight a concerning decline in knee stability over time associated with Leeds-Keio grafts. For comparative visual summaries, refer to [Figure 2].

#### Functional Outcomes

Leeds-Keio synthetic grafts provided mixed but generally satisfactory long-term functional outcomes in ACL reconstruction. Despite limited mechanical durability, these grafts yielded moderate-to-high subjective satisfaction and functional recovery. Lysholm scores improved from a preoperative mean of 62 to postoperative means of 87 at six months, peaking at 90 at one year, and stabilizing at 87 at five years. Approximately 80% of patients achieved satisfactory Lysholm scores, with IKDC classifications rated as ‘excellent’ or ‘good’ in 54% of cases [13], [32], [35], [Figure 3], [Figure 4]. Subjective IKDC scores reflected moderate success: 6% of patients rated their knee as ‘normal’, 28% as ‘nearly normal’, while 67% still considered their knee ‘abnormal’ or ‘severely abnormal’ at a long-term follow-up [34], [Figure 7]. In contrast, objective IKDC assessments revealed poorer outcomes, with 0% of patients classified as Grade A, 6% as Grade B, and 94% as Grade C or D, thus suggesting persistent mechanical laxity despite reasonable subjective satisfaction [34]. Overall patient satisfaction remained high: approximately 90% of patients reported being satisfied or highly satisfied with the procedure, and 92% returned to activity levels exceeding their preoperative baseline. However, only 40% maintained their pre-injury activity levels beyond ten years [30], [33], [34]. Tegner activity scores increased from a preoperative mean of 3.0 to 5.5 at five years but declined slightly to a mean of 4.67 at 13.3 years, reflecting a gradual reduction in physical activity over time [34], [Figure 5]. Return-to-sport outcomes were generally favorable: 87.5% of patients resumed athletic activity, of which, 45% were amateur athletes, 42.5% recreational, and 12.5% did not return to sport [35], [Figure 6]. Despite encouraging subjective and return-to-activity results, a notable gap persisted between perceived recovery and objective mechanical stability. Up to one-third of patients reported ongoing instability or discomfort during high-demand activities [31], [32], [33]. These findings suggest that while Leeds-Keio grafts may support functional performance in lower-demand populations, they are susceptible to mechanical deterioration over time, particularly in athletic or high-impact environments.

### GORE-TEX

#### Failure Rates

GORE-TEX synthetic grafts, typically used in younger, physically active individuals (mean age 25 years, mean Tegner score 7.3), have raised significant concerns regarding long-term durability. Reported graft rupture rates range between 12% and 33%, with failure mechanisms involving both trauma and progressive degenerative changes [36], [37]. One large cohort study (n = 188) reported a 12% rupture rate occurring at a mean of 26.2 months postoperatively, most often at the femoral insertion site. Nearly half of these patients experienced prior joint effusion, and 26% exhibited graft loosening on KT-1000 testing before rupture occurred [36]. In another long-term study, a failure rate of 33% was observed at a ≥3-year follow-up. Only 27% of these failures were trauma-related, while the remainder were attributed to progressive graft attrition, likely from intra-articular fraying or notch impingement [37]. Kaplan–Meier analyses demonstrated a steep decline in survival: from 82% at 48 months to 44% at 62 months [37]. Additional mechanical concerns included loosening (>3 mm anterior translation in 34% of patients), sterile joint effusions, and a 4% explanation rate due to recurrent synovitis [36]. Collectively, these findings suggest a mean GORE-TEX graft failure rate of approximately 22.5%, with a wide spectrum of complications including rupture, elongation, synovitis, and biomechanical insufficiency. These results underscore the limited long-term reliability of GORE-TEX grafts in ACL reconstruction, particularly in high-demand populations [Figure 1].

#### Knee Stability

Postoperative knee stability initially improved significantly following ACL reconstruction with GORE-TEX grafts. KT-1000 arthrometer measurements showed a reduction in anterior laxity from 4.3 mm to 1.8 mm in acute cases and from 6.0 mm to 2.3 mm in chronic cases [38]. Clinical assessments corroborated these findings, with 87% of patients achieving grade 0 or 1+ stability on anterior drawer and Lachman tests, and 85% demonstrating similar results on pivot-shift testing [38]. However, long-term stability outcomes were more variable. Instrumented laxity testing revealed that while 67% of patients maintained stable laxity (<3 mm), 22% developed significant instability (>5 mm). Graft loosening of ≥3 mm was identified in 34% of cases and was strongly associated with symptomatic instability, higher pivot-shift grades, and diminished clinical performance. These findings were especially prominent in cases of a graft failure, where increased anterior laxity and subjective knee instability were frequently reported [36], [37]. Despite these mechanical concerns, 88% of patients subjectively reported improved knee function at follow-up [36]. Overall, while short-term postoperative stability with GORE-TEX grafts appears promising, notable long-term deterioration remains a significant concern. For comparative visual summaries, refer to [Figure 2].

#### Functional Outcomes

GORE-TEX synthetic ligaments demonstrated strong subjective functional improvements following ACL reconstruction, though long-term athletic performance was often limited. Postoperative Lysholm scores averaged 92 overall, with acute cases performing slightly better than chronic ones (93.5 vs. 92), thus indicating high levels of self-reported knee function across patient subgroups [38], [Figure 3], [Figure 4]. Symptom relief was significant: reports of knee instability (‘giving way’) declined from 64.1% preoperatively to 5.1% postoperatively; pain prevalence dropped from 92.3% to 25.6%; and knee swelling decreased from 61.5% to 15.5%. Additional improvements were noted in locking symptoms and stair-climbing ability as 94.9% of patients could ascend stairs comfortably after surgery, compared to 56.4% prior to the procedure. Overall, approximately 90% of patients subjectively rated their knee as significantly improved postoperatively [38]. Despite these favorable subjective outcomes, long-term athletic performance remained constrained. Tegner activity scores were unchanged in 89% of patients, decreased in 10%, and improved in only 1%, thus reflecting a limited return to high-impact or competitive activity [36], [Figure 5], [Figure 6]. This contrast between symptom resolution and activity resumption suggests that while GORE-TEX grafts are effective in restoring the everyday knee function, they are less suited for supporting sustained athletic performance in physically demanding populations. IKDC outcomes are summarized in [Figure 7].

## Discussion

This review compared the clinical performance of biological grafts – bone-patellar tendon-bone (BPTB), hamstring tendon (HST), and quadriceps tendon (QT) – with synthetic options including LARS, Leeds-Keio, and GORE-TEX in anterior cruciate ligament (ACL) reconstruction. Across the available evidence, biological autografts demonstrated more consistent long-term success, with lower rupture rates, superior mechanical stability, and better histological integration into host tissue. In contrast, synthetic grafts – while enabling faster early rehabilitation and avoiding donor-site morbidity – were associated with higher complication rates, inferior biological incorporation, and poorer survivorship over time [8], [10], [12], [13], [23], [24], [39].

### Graft Failure and Durability

Among biological options, BPTB autografts consistently achieved the lowest failure rates, with large series reporting survival rates exceeding 90% and revision rates ranging between a 1% and 4% [8], [10], [11], [12]. QT grafts offered similarly favorable durability, with failure rates typically under 4%, and showed reduced donor-site morbidity, which makes them an increasingly preferred alternative [12], [21]. HST grafts performed well overall but demonstrated greater vulnerability to failure in high-demand or younger populations, particularly when graft diameters were <8 mm or when femoral tunnel widening occurred [15], [17]. The primary causes of biological graft failure were typically traumatic re-injury or biomechanical overload.

In contrast, synthetic grafts demonstrated a broader and significantly higher range of failure rates. Leeds-Keio grafts failed in up to 47% of cases at 5-year follow-up [33], and GORE-TEX grafts had failure rates up to 33%, often occurring in the absence of trauma and due instead to progressive mechanical degradation and material fatigue [36], [37]. Even LARS, which initially performed favorably in selected populations, showed increasing rates of rupture, elongation, or reoperation by 5–6 years postoperatively, particularly in active patients [22], [24], [29].

Synthetic graft failure is multifactorial: mechanically, synthetic fibers are susceptible to creep, fraying, and abrasion, especially when the graft isometry is imperfect. Biologically, they lack the capacity for intrinsic remodeling and vascularization seen in autografts. Instead, they often elicit chronic *Polyethylene Terephthalate* (PET)-based materials such as LARS and GORE-TEX [33], [39], [40]. These histological findings help explain why synthetic grafts often deteriorate progressively even without an inciting injury.

### Early vs. Late Complications

A clearer distinction between early and late complications is essential to understanding the graft performance. Early postoperative issues, such as infection, hemarthrosis, effusion, and joint stiffness, occur in both biological and synthetic reconstructions and are predominantly related to the surgical technique, tunnel placement, and rehabilitation protocols [5], [6], [18], [19]. These are generally preventable and not intrinsically linked to the graft material.

In contrast, late complications differ substantially by the graft type. Biological grafts may develop tunnel widening, graft rupture, or laxity, often due to trauma or biomechanical overloading. Their failure typically reflects technical factors (e.g., malpositioned tunnels), an insufficient graft size, or reinjury. Synthetic grafts, on the other hand, are more prone to late failure due to intrinsic material fatigue, micromotion, or chronic biological responses. Graft elongation, fraying and synovitis are common, particularly in Leeds-Keio and GORE-TEX devices, where long-term failure can occur without any specific traumatic event [33], [34], [36]. Some reports also document accelerated osteoarthritis and graft rejection, especially in older synthetic models.

### Knee Stability and Function

In terms of mechanical performance, both BPTB and QT autografts demonstrated superior postoperative stability, with >95% of patients achieving Grade 0–1 laxity or Lachman and pivot shift testing across multiple studies [7], [8], [12], [22]. HST grafts also produced acceptable outcomes, although a higher percentage of patients exhibited residual laxity of >3 mm of KT-1000 testing, particularly in cases involving smaller graft diameters or revision procedures [15], [16]. Still, most studies reported satisfactory functional outcomes in these patients.

Synthetic grafts, particularly LARS, initially provided excellent short-term stability. KT-1000 measurements showed postoperative anterior tibial translation reductions to <2mm in most cases [22], [24]. However, this early stability often deteriorated over time. Several cohorts reported progressive stretching or elongation of the synthetic material, with a notable rise in side-to-side differences and return of pivot shift signs by year 5–6 [23], [33]. Leeds-Keio grafts showed a marked decline in stability over time, with KT-1000 values exceeding 10 mm in up to 32% of cases at a 5-year follow-up [33], and GORE-TEX reconstructions showed increasing rates of mechanical loosening and instability after 2–3 years [36], [37].

Functional outcomes mirrored this pattern. Autografts, especially BPTB and QT, consistently achieved Lysholm scores ≥90 in a long-term follow-up, along with high rates of return to pre-injury sports levels [9], [12], [20]. Tegner scores typically declined modestly but remained within the recreational-to-competitive range. HST grafts yielded slightly lower functional scores, particularly in revision cases or high-impact athletes, but still demonstrated good overall patient satisfaction [15], [16]. Synthetic grafts often showed high early subjective satisfaction (Lysholm >90; IKDC Grade A/B in 90–99%), yet return-to-sport rates were lower, particularly in high-performance athletes [24], [28], [29]. In LARS and Leeds-Keio groups, only 31–50% of patients resumed their pre-injury Tegner level, and long-term functional decline was commonly linked to recurrent laxity or mechanical graft degradation [33], [34].

### Osteoarthritis and Tunnel Changes

*Osteoarthritis* (OA) progression after ACL reconstruction is multifactorial, influenced by the patient age, meniscal status, cartilage integrity, and rehabilitation protocols. However, the graft type may further modulate the OA risk. Biological grafts, and particularly BPTB and QT, do not appear to accelerate OA beyond the expected natural history following ACL injury [15], [16]. In contrast, synthetic grafts, especially Leeds-Keio, were associated with a markedly higher prevalence of radiographic OA at long-term follow-up. One series found degenerative changes in 100% of operated knees after Leeds-Keio reconstruction, versus just 39% of contralateral knees [34].

Tunnel widening also differed by the graft type. HST grafts exhibited more pronounced tunnel expansion, likely due to their soft-tissue-to-bone integration and lower initial fixation stiffness [17]. BPTB and QT autografts, with bone-to-bone healing interfaces, demonstrated reduced tunnel enlargement and more stable long-term fixation [12], [42]. Synthetic grafts, particularly GORE-TEX and Leeds-Keio, showed greater tunnel widening, likely driven by graft micromotion, polyethylene debris generation, and persistent inflammatory responses.

### Histological Integration

Histologic studies underscore the limitations of synthetic grafts compared to autografts. Biological grafts undergo progressive ‘ligamentization’, involving cellular infiltration, neovascularization, and collagen remodeling. These processes allow the autograft to biologically integrate and adapt to *in vivo* loading over time [16], [22]. In contrast, synthetic grafts often demonstrate persistent acellularity, poor vascularization, and chronic inflammatory responses. Microscopic findings frequently reveal polyethylene terephthalate (PET) fiber debris surrounded by foreign-body giant cells, chronic synovitis, and fibrous encapsulation rather than ligamentous transformation [33], [39], [40], [41]. These histological limitations correlate with mechanical degradation, recurrent instability, and joint deterioration seen in long-term synthetic graft follow-up.

### Clinical Implications

Taken together, the evidence supports BPTB and QT autografts as the most reliable choices for ACL reconstruction, especially in younger, active patients who demand long-term mechanical integrity and functional return [8], [9], [10], [12], [18], [20]. QT grafts may be preferable in patients at risk of anterior knee pain or kneeling discomfort, as they demonstrate lower rates of anterior knee pain and donor-site morbidity compared to BPTB [12], [18], [20]. BPTB remains the gold standard for high-demand athletic cases, showing superior return-to-sport rates and consistent long-term mechanical durability [8], [9], [10]. HST autografts remain viable in selected populations, particularly when preservation of the extensor mechanism is important, provided that the adequate graft diameter is ensured to reduce the failure risk [10], [15], [16], [17].

Synthetic grafts may retain a limited role in specific clinical scenarios. For instance, LARS has been used in multi-ligament reconstructions or in patients where autograft harvest is contraindicated due to previous procedures or comorbidities [22], [23], [28]. However, these indications must be weighed against- well-documented mid- to long-term complications, including mechanical failure, graft loosening, synovitis, and secondary osteoarthritis [24], [33], [34], [36], [37], [38]. Graft selection should therefore be individualized based on patient-specific factors such as the activity level, anatomical considerations, graft size, and tolerance for donor-site morbidity.

## Limitations

This review is limited by variability in follow-up durations, outcome definitions, and surgical techniques across the included studies. Differences in the failure criteria (e.g., subjective instability vs. instrumented laxity vs. revision surgery) limit direct comparisons. Additionally, while numerical values were pooled to represent the average outcomes, these data were synthesized from heterogenous cohorts and not from a formal meta-analysis. As such, findings should be interpreted cautiously and viewed as descriptive rather than definitive.

## Conclusion

This review highlights the clinical superiority of biological grafts, particularly bone-patellar tendon-bone (BPTB) and quadriceps tendon (QT) autografts, for anterior cruciate ligament (ACL) reconstruction. These grafts consistently demonstrate lower failure rates, superior mechanical stability, and more favorable long-term functional outcomes compared to synthetic alternatives. While hamstring tendon (HST) autografts also yield satisfactory results in selected populations, synthetic grafts such as LARS, Leeds-Keio, and GORE-TEX are associated with higher complication rates, limited biological integration, and a greater risk of long-term joint degeneration.

Current evidence supports BPTB and QT autografts as the most reliable options for restoring long-term knee function, particularly in younger, active patients. Synthetic grafts may still have a role in specific clinical scenarios, such as multi-ligament injuries or in patients with contraindications to autograft harvest, but their use requires caution given the associated risks. Graft selection should ultimately be individualized, based on patient activity level, anatomical factors, and tolerance for donor-site morbidity. Ongoing research is warranted to improve the biocompatibility of synthetic materials and to accelerate the development of tissue-engineered grafts that offer both mechanical strength and sustained biological integration.

## Data Availability

No new data were created or analyzed in this study. Data sharing is not applicable to this article.

## Abbreviations

ACL – Anterior Cruciate Ligament

BPTB – Bone-Patellar Tendon-Bone

QT – Quadriceps Tendon

HST – Hamstring Tendon

LARS – Ligament Advanced Reinforcement System

IKDC – International Knee Documentation Committee

OA – Osteoarthritis

RCT – Randomized Controlled Trial

AM – Anteromedial

PL – Posterolateral

BMI – Body Mass Index

MRI – Magnetic Resonance Imaging

KT-1000/2000 – Knee Testing Arthrometer

PROMs – Patient-Reported Outcome Measures

PET – Polyethylene Terephthalate

SF-36 – Short Form 36 Health Survey
